# On the key role of droughts in the dynamics of summer fires in Mediterranean Europe

**DOI:** 10.1038/s41598-017-00116-9

**Published:** 2017-03-06

**Authors:** Marco Turco, Jost von Hardenberg, Amir AghaKouchak, Maria Carmen Llasat, Antonello Provenzale, Ricardo M. Trigo

**Affiliations:** 1 Department of Applied Physics, University of Barcelona, Barcelona, 08028 Spain; 2 Earth Science Department, Barcelona Supercomputing Center, Barcelona, 08028 Spain; 3 Institute of Atmospheric Sciences and Climate (ISAC), National Research Council (CNR), Torino, 10133 Italy; 4 Center for Hydrometeorology and Remote Sensing, Department of Civil and Environmental Engineering, University of California, Irvine, CA 92697 USA; 5 Institute of Geosciences and Earth Resources (IGG), National Research Council (CNR), Pisa, 56124 Italy; 6 Instituto Dom Luiz (IDL), Faculdade de Ciências, Universidade de Lisboa, Lisboa, 1749-016 Portugal

## Abstract

Summer fires frequently rage across Mediterranean Europe, often intensified by high temperatures and droughts. According to the state-of-the-art regional fire risk projections, in forthcoming decades climate effects are expected to become stronger and possibly overcome fire prevention efforts. However, significant uncertainties exist and the direct effect of climate change in regulating fuel moisture (e.g. warmer conditions increasing fuel dryness) could be counterbalanced by the indirect effects on fuel structure (e.g. warmer conditions limiting fuel amount), affecting the transition between climate-driven and fuel-limited fire regimes as temperatures increase. Here we analyse and model the impact of coincident drought and antecedent wet conditions (proxy for the climatic factor influencing total fuel and fine fuel structure) on the summer Burned Area (BA) across all eco-regions in Mediterranean Europe. This approach allows BA to be linked to the key drivers of fire in the region. We show a statistically significant relationship between fire and same-summer droughts in most regions, while antecedent climate conditions play a relatively minor role, except in few specific eco-regions. The presented models for individual eco-regions provide insights on the impacts of climate variability on BA, and appear to be promising for developing a seasonal forecast system supporting fire management strategies.

## Introduction

Most of the total burned area (BA) in Europe occurs in Mediterranean regions during summer, with an average of about 4500 *km* ^2^/*yr* ^1^. These fires cause extensive economic and ecological losses, and even human casualties^2^. The Mediterranean region is located in a transition area under the alternate influence of sub-tropical and mid-latitude climates^3^. Here, ecosystems and human societies are strongly impacted by frequent weather-driven natural hazards, such as droughts^4, 5^, heat waves^6, 7^, and wildfires^8^, all of which are expected to increase in frequency and severity under climate change^9–11^. Furthermore, concurrent drought-heatwave events have increased substantially in the past decades, and are expected to increase further in a warming climate^12^.

Forest fires are a complex natural process associated with factors of different origin, such as climate and weather, human activities and vegetation conditions^13^. Although anthropogenic ignition is dominant in most Mediterranean regions^14^, variations in the ease of ignition and in the conditions affecting fire after ignition are mainly governed by the presence, amount and connectivity of fuel (fuel amount) and its moisture content (fuel moisture), which in turn depend on climate variability^13^. The propagation of forest fires is further controlled by wind conditions^15, 16^.

The link between climate and fire is often analysed under the intermediate fire-productivity hypothesis^17–19^, which suggests that fire activity reaches two minimums, one dominated by high aridity values where fire spread is mostly limited by the fuel amount, and another characterised by low aridity where fuels are abundant and fires are mainly limited by the fuel moisture content. In fuel-limited ecosystems, antecedent wet conditions may regulate the fuel amount and its structure, while in rarely dry ecosystems with abundant fuel, droughts and hot spells can influence fuel dryness^20^. Mediterranean-type ecosystems can be considered “intermediate” ecosystems, where both fuel moisture and fuel structure can play a role in shaping fire regimes^18, 20^.

Several previous works suggest that coincident drought conditions and high temperatures promote larger fires in southern Europe^21–23^, Portugal^24, 25^, the Spanish Mediterranean regions^26–28^, the entire Iberian Peninsula^29, 30^, southern France^16, 31^, Italy^32^ and Greece^33^. The drought-driven impacts of fire may be across multiple time scales, although these are generally poorly quantified. The existing studies support the view that fires are related also to antecedent climate variables in some Mediterranean environments. For example, year-to-year changes are negatively correlated with concurrent summer rainfall and positively correlated with antecedent (2 years) summer rainfall in a Mediterranean region (Valencia, eastern Spain^27^). Similarly, analysing the relationships between forest fire activity and meteorological variables in southern France^16^, Greece^33^ and northeastern Spain^28^ reveals significant correlations with both fire-season and lagged climate variables. However, a full picture of the possible links between antecedent climate and fire activity for the whole of Mediterranean Europe is still missing.

Studies on the sensitivity of Mediterranean forest fires at continental scale are indeed rare. A recent study^34^ explored the relationship between above-normal wildfire activity and meteorological droughts, using the Standardised Precipitation Index (SPI^35^) and aggregating the data over the whole of Mediterranean Europe as well as over two sub-regions: the Iberian Peninsula, and southern Italy and Greece taken together. The study indicated that above-normal summer wildfire activity could be predicted several months in advance by taking into account the effect of drought on fuel dryness. In this case, the focus was on exploring predictive relationships between fires and meteorological drought represented by the SPI. Although the authors did not specifically analyse the link between fire and antecedent climate variables, their results showed that antecedent meteorological conditions could be a source of predictability of regional wildfire activity^34^. Nevertheless, as the authors noted, two main limitations of their approach were (i) the sub-continental scale of the assessment (the whole of Mediterranean Europe and two sub-regions, the Iberian Peninsula, and the region of southern Italy and Greece taken together), which prevent local wildfire management, and (ii) the fact that the availability of burnable biomass was not taken into account. We point out that another major caveat of the SPI is its insensitivity to heat or temperature-related variables.

Several recent studies show that droughts are compounded with prolonged high temperatures^36, 37^. As such, the SPI is not particularly appropriate for applications where both precipitation and temperature are important, since it takes into account neither the increase in temperature in the Mediterranean observed in recent decades^3, 7, 38^, nor the much larger temperature increase expected under climate change scenarios^6, 30^. The Standardized Precipitation Evapotranspiration Index (SPEI^39^), which is similar to the SPI, has the advantage of allowing analysis of multiple temporal scales (as it is typically computed over accumulation times from 1 month to 12 months), and the additional advantage of including the effects of temperature variability on drought assessment^39^. The summer SPEI has been shown to capture the drought impacts on hydrological, agricultural, and ecological variables (e.g. refs 40–43) better than other indices such as SPI or the Palmer drought severity index.

Quantifying the spatial and temporal variability of the impacts of drought and antecedent wet conditions on fires is also crucial to providing additional insight on management strategies. Specifically, the time scales (i.e. drought duration) and timing (i.e. when droughts occur) which give drought and/or antecedent wet conditions their strongest influence on fires are yet to be assessed across Mediterranean Europe. This information would greatly help policy-makers and civil protection agencies, improving early warning systems and allowing more efficient fire management strategies. Furthermore, assessing the spatial pattern of drought/wet sensitivity would provide relevant information on the mechanisms linking climate, fires and Mediterranean ecosystems.

To sum up, the development of predictive relationships between fires and coincident drought and antecedent climate anomalies at regional scale in Mediterranean Europe remains to be studied. This is mainly due to limitations in observations, difficulties in disentangling the many drivers of forest fires, and ultimately in translating drought-fire relationships into useful information. A comprehensive assessment of the climate-fire lagged relationships, as discussed in this work, can help narrow this gap. To address these issues we use a large, high-quality database provided by the European Forest Fire Information System (EFFIS^44^) to analyse the impact of coincident drought and antecedent wet conditions on summer BA in Mediterranean Europe (Portugal, Spain, southern France, Italy, and Greece), modelling the interaction between the SPEI and BA. Specifically, our main goal is to answer the following research questions:

1. To what extent is the variability in BA related to drought and antecedent climate conditions?
2. How do these relationships vary geographically and in different ecological regions?
3. What time window of drought/antecedent wet periods promotes larger fires?
4. Can we model year-to-year variations of BA with parsimonious drought-fire models?

## Results

**Figure 1:**
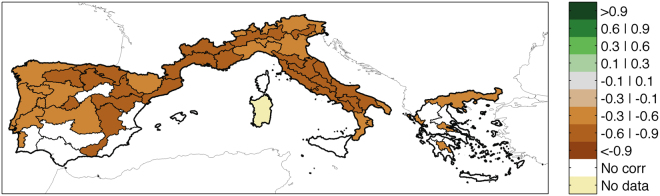
Correlations between detrended log(*BA*) and *SPEI*_3_(0, 8), the SPEI for an accumulation time scale of 3 months and calculated in August (8) of the coincident summer (i.e. with the time lag of 0 year). Only correlations that are collectively significant from an FDR test^45^ are shown. This figure is created with Matlab version R2012a (http://www.mathworks.com/).

To identify the key drought variables potentially affecting the Burned Area we use an empirical approach and systematically explore cross-correlations between detrended drought variables and fires for each eco-region in Mediterranean Europe (see Supplementary Fig. S1). Figure 1 shows an illustrative example of the correlation between the log(*BA*) series and the coincident *SPEI*_3_(0, 8), i.e., considering 3 months of accumulation time and calculated in August (8) of the same fire year (0). Most of the analysed regions show statistically significant negative correlations. Since negative SPEI values correspond to hot and dry conditions, the negative *BA* − *SPEI*_3_(0, 8) correlations unsurprisingly indicate that hotter and drier conditions in June, July, and August led to a larger Burned Area for the same summer. In order to find the time scale (we consider 3, 6 or 12 months of aggregation) and the period in which BA is driven by coincident drought conditions (we consider same summer and previous spring drought values as potential candidates), we seek the maximum correlations (in absolute value) across the pool of all correlation values (see Supplementary Fig. S2).

**Figure 2:**
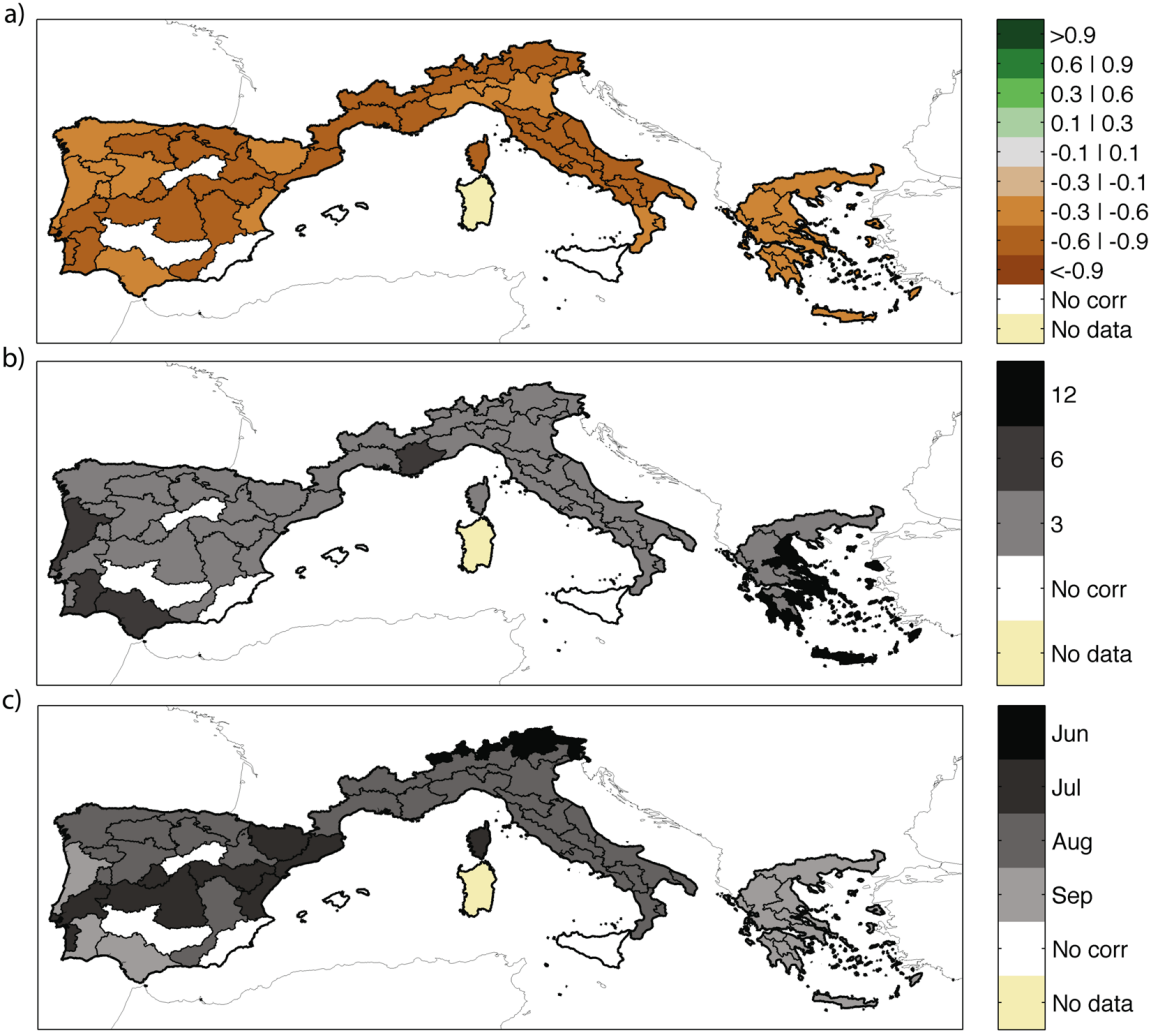
(**a**) Maximum significant correlation (in absolute value) between detrended log(*BA*) and SPEI; (**b**) length of the period (3, 6 and 12 months) and (**c**) final month of accumulation of the SPEI for which the absolute value of the correlation is maximum. Only correlations that are collectively significant from an FDR test^45^ are shown. This figure is created with Matlab version R2012a (http://www.mathworks.com/).

Figure 2a shows the maximum correlations (in absolute value) between log(*BA*) and the SPEI for the different accumulation months (Fig. 2b) and periods of the year (Fig. 2c) for which the index is calculated. Only a few regions (1 in central Spain, 2 in southern Spain and Sicily) do not show any links with coincident drought. Generally, the strongest correlations are obtained for the time scale of 3 months (Fig. 2b), although some regions in Portugal, southern Spain, southern France and Greece reveal stronger correlations at longer accumulation times. In most of Spain, southern France and Italy, the highest (in absolute value) correlations are observed for the SPEI calculated in August (Fig. 2c). For Portugal, a mixed timing (June to August) is observed, while Greece displays the strongest correlation in September. Taken together, these results indicate that same-summer drought conditions generally control the area burned by fires.

**Figure 3:**
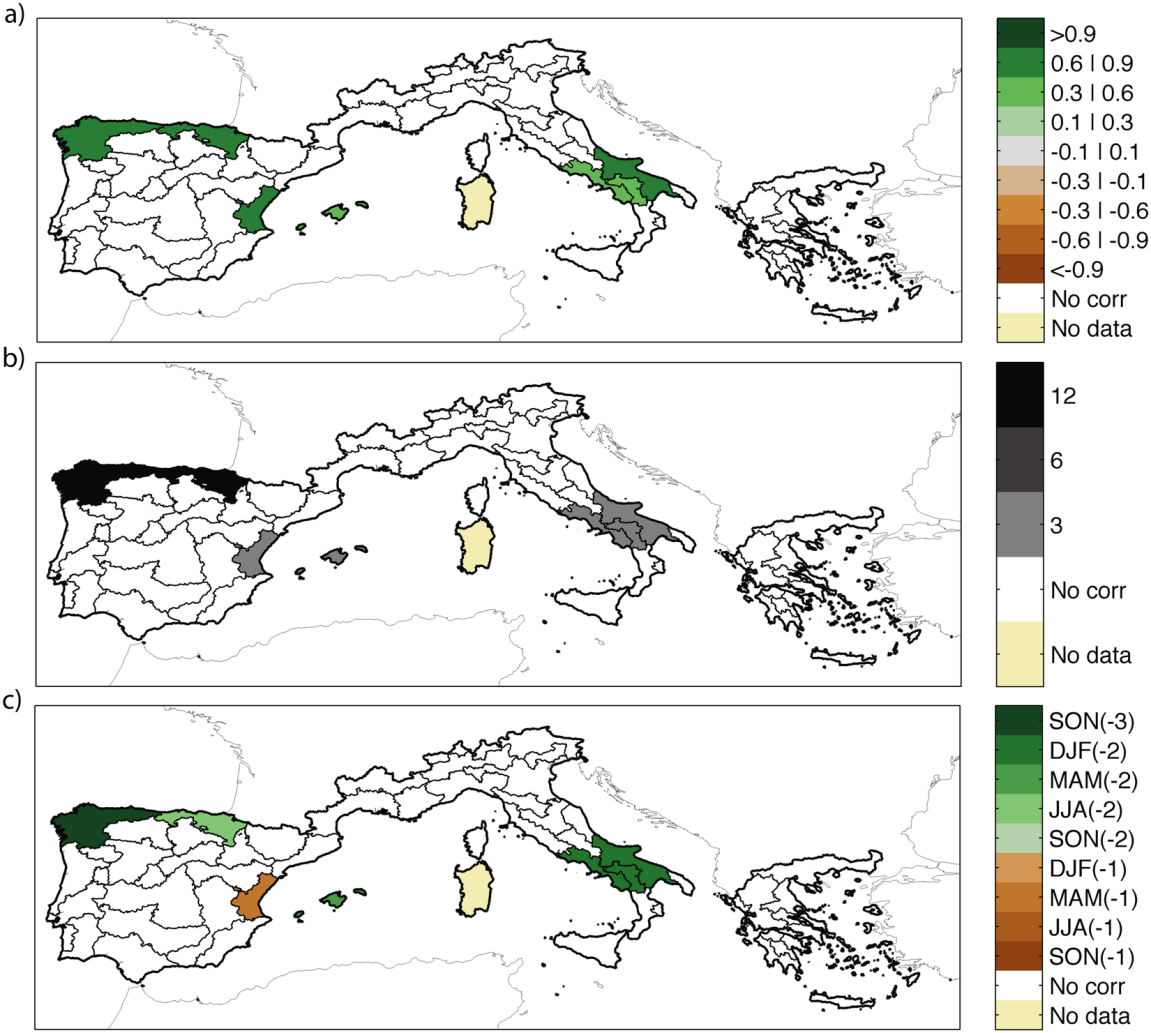
(**a**) Maximum significant partial correlations between detrended log(*BA*) and SPEI; (**b**) accumulation time scale (3, 6 and 12 months) of the SPEI; (**c**) seasons when the antecedent SPEI is calculated. Only correlations that are collectively significant from an FDR test^45^ are shown. This figure is created with Matlab version R2012a (http://www.mathworks.com/).

The identification of the indicators for antecedent climate effects is subtler. To find the key antecedent time scale, we calculate the partial correlation between log(*BA*) and the antecedent SPEI, controlling for the coincident drought variables obtained in the previous step and depicted in Fig. 2. For those regions where no coincident drought variable is found, the standard correlation is tested. We analyze SPEI values for different periods, ranging from 45 to 0 months before the end of the fire season (i.e. from January of 3 years before the fire season to September of the same year of the fire season), and for different accumulation times (3, 6 and 12 months). For each region, we obtain 138 correlation values (the 3 accumulation time scales and the 46 different months to which the index refers; see Supplementary Fig. S2). Then we identify the maximum partial correlation values between BA and the 138 different drought index values. Figure 3a reveals that only 7 eco-regions show significant positive correlations between the SPEI and BA, with a variety of accumulation time scales and antecedent seasons found for the different areas. The highest correlations are obtained for the time scale of 3 and 12 months (Fig. 3b), and generally for a lag time of 2 years before the fire season (Fig. 3c). In northwestern Spain, wet conditions in the autumn of 3 years before also play a role, while in eastern Spain the relevant antecedent climatic conditions are those of one year before the fire season. In all these regions, we can conclude that also antecedent wet periods have an influence on fires, presumably because fine-fuel productivity is controlled by climate variability prior to wildfire events.

Owing to the large number of repetitions in the correlation tests (considering different accumulation periods, time-steps and regions), we can expect several correlations to appear significant just by chance, i.e. even if BA is independent of droughts/antecedent wet periods. We address the problem of multiple comparisons with a False Discovery Rate (FDR) test^45^. We apply the test on the p-values of the (partial) correlations and conservatively set a false rejection rate of *q* = 0.05. From this test, considering the coincident drought conditions, 35% of the correlations with a p-value < 0.05 is not collectively significant, while for the antecedent effect the percentage is 92%. That is, a large fraction of individually significant relationships are neglected in order to avoid false positives.

**Figure 4:**
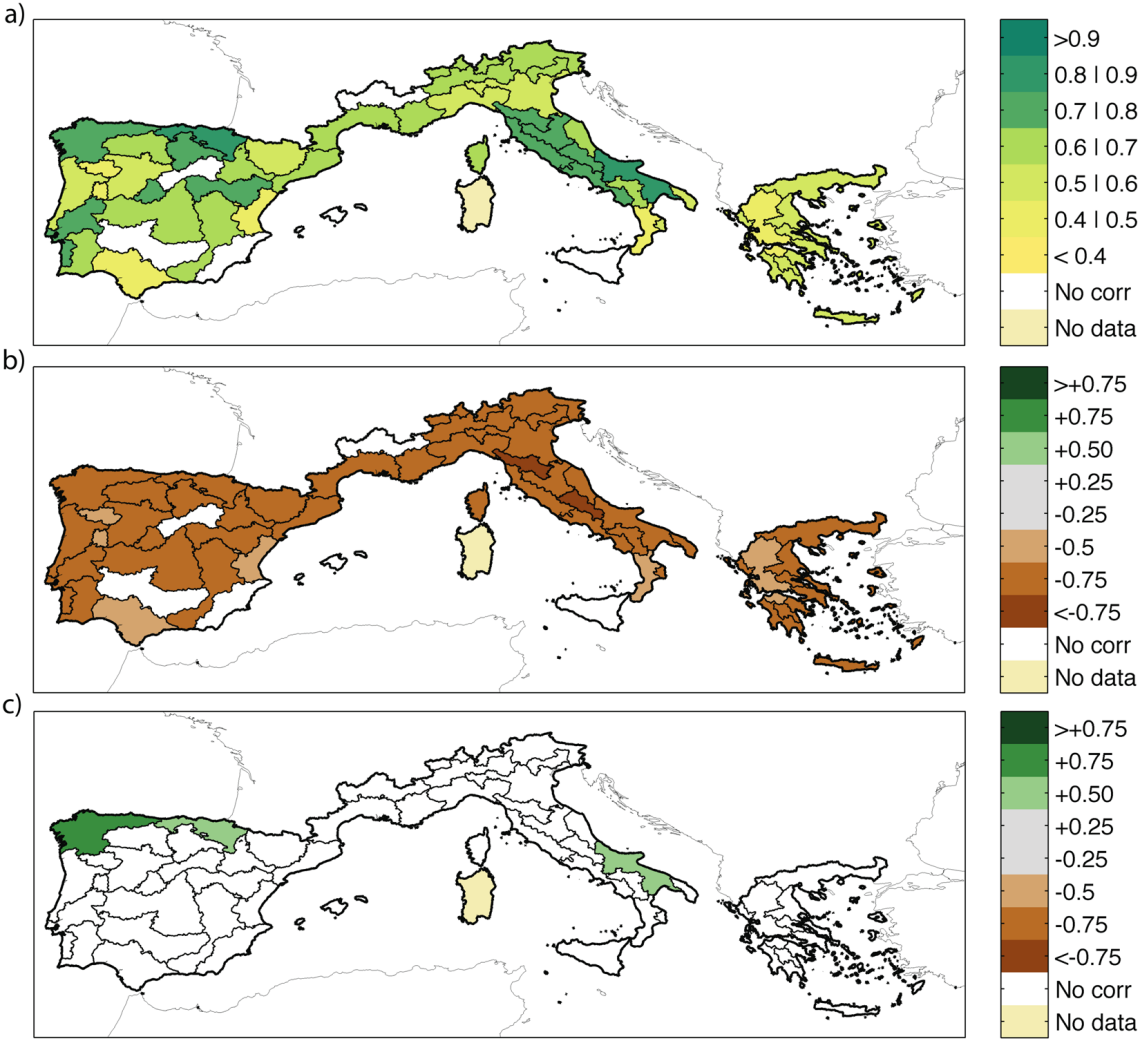
MLR results: (**a**) correlation between modelled and observed log(*BA*) (detrended) for each eco-region; (**b**) coefficient weights for the coincident drought conditions (SPEI); (**c**) coefficient weights for the antecedent drought conditions (SPEI). This figure is created with Matlab version R2012a (http://www.mathworks.com/).

The key variables identified above can be considered potential predictors for developing Multiple Linear Regression models (hereafter, MLR) for each individual eco-region. Figure 4a summarizes the skill of the MLR models and their parameters for each eco-regions of the Mediterranean Europe (see Table 1 for an exact definition of the model for each region). The average of the correlations of all models is 0.63. Note that these models are in-sample estimates, i.e., hindcasts. An important test of these models is to verify their ability to perform out-of-sample predictions of BA from the knowledge of predictor data outside the period used to train the model. The correlations of the out-of-sample predictions (obtained through a leave-one-out cross-validation) with the data indicate a good model skill also in prediction mode, with the average correlation value at 0.58 (see the final column of Table 1).

**Table 1:** Empirical SPEI-fire models (Eq. 1) for each eco-region (labelled according to Fig. S1) and the correlation for the reconstruction model (RhoIN: in-sample) and for the leave-one-out cross validation model (RhoOUT: out-of-sample).

Region	Model	RhoIN	RhoOUT
ES01	*Y* = −0.52 · *SPEI*_3_(0, 8) + 0.53 · *SPEI*_12_(3, 11)	0.77	0.71
ES02	*Y* = −0.70 · *SPEI*_3_(0, 8) + 0.43 · *SPEI*_12_(2, 8)	0.86	0.84
ES03	*Y* = −0.62 · *SPEI*_3_(0, 8)	0.62	0.53
ES04	*Y* = −0.74 · *SPEI*_3_(0, 8)	0.74	0.69
ES05	*Y* = −0.57 · *SPEI*_3_(0, 8)	0.57	0.45
ES07	*Y* = −0.72 · *SPEI*_3_(0, 7)	0.72	0.68
ES08	*Y* = −0.61 · *SPEI*_3_(0, 7)	0.61	0.55
ES10	*Y* = −0.44 · *SPEI*_6_(0, 9)	0.44	0.40
ES11	*Y* = −0.70 · *SPEI*_3_(0, 8)	0.70	0.67
ES13	*Y* = −0.45 · *SPEI*_3_(0, 7)	0.45	0.37
ES14	*Y* = −0.69 · *SPEI*_3_(0, 7)	0.69	0.64
ES15	*Y* = −0.53 · *SPEI*_3_(0, 7)	0.53	0.47
ES17	*Y* = −0.63 · *SPEI*_3_(0, 8)	0.63	0.59
FR01	*Y* = −0.61 · *SPEI*_3_(0, 8)	0.61	0.54
FR03	*Y* = −0.64 · *SPEI*_6_(0, 8)	0.64	0.58
FR04	*Y* = −0.63 · *SPEI*_3_(0, 7)	0.63	0.60
GR01	*Y* = −0.54 · *SPEI*_3_(0, 9)	0.54	0.48
GR02	*Y* = −0.50 · *SPEI*_3_(0, 9)	0.50	0.43
GR03	*Y* = −0.59 · *SPEI*_12_(0, 9)	0.59	0.52
IT03	*Y* = −0.52 · *SPEI*_3_(0, 8)	0.52	0.48
IT04	*Y* = −0.43 · *SPEI*_3_(0, 8)	0.43	0.36
IT05	*Y* = −0.66 · *SPEI*_3_(0, 8)	0.66	0.62
IT06	*Y* = −0.70 · *SPEI*_3_(0, 8)	0.70	0.67
IT07	*Y* = −0.74 · *SPEI*_3_(0, 8) + 0.42 · *SPEI*_3_(3, 12)	0.87	0.84
IT08	*Y* = −0.76 · *SPEI*_3_(0, 8)	0.76	0.73
IT09	*Y* = −0.66 · *SPEI*_3_(0, 8)	0.66	0.62
IT10	*Y* = −0.74 · *SPEI*_3_(0, 8)	0.74	0.71
IT11	*Y* = −0.57 · *SPEI*_3_(0, 8)	0.57	0.54
IT12	*Y* = −0.77 · *SPEI*_3_(0, 8)	0.77	0.75
IT13	*Y* = −0.74 · *SPEI*_3_(0, 8)	0.74	0.73
IT14	*Y* = −0.53 · *SPEI*_3_(0, 8)	0.53	0.49
IT15	*Y* = −0.66 · *SPEI*_3_(0, 8)	0.66	0.62
IT16	*Y* = −0.69 · *SPEI*_3_(0, 6)	0.69	0.66
PT01	*Y* = −0.63 · *SPEI*_6_(0, 9)	0.63	0.58
PT02	*Y* = −0.71 · *SPEI*_3_(0, 7)	0.71	0.66
PT03	*Y* = −0.52 · *SPEI*_6_(0, 9)	0.52	0.43
PT04	*Y* = −0.45 · *SPEI*_3_(0, 8)	0.45	0.36

**Figure 5:**
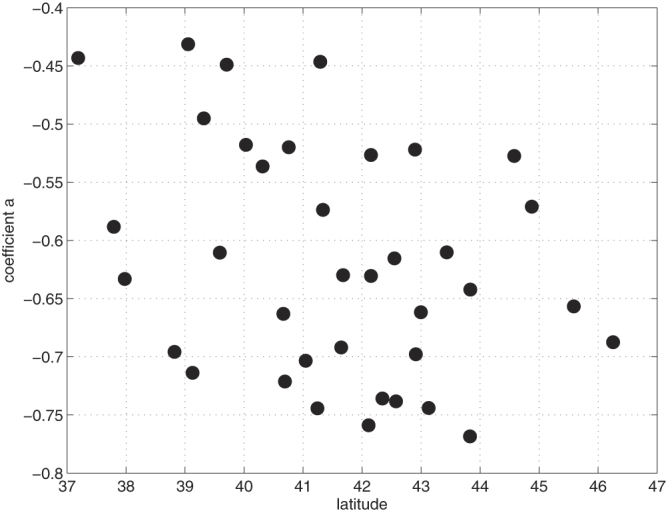
Weights of the CD parameters of the SPEI-BA model (i.e. the coefficients a(i) of Eq. 1) plotted against the latitude of the centroids of the eco-regions.

Finally, this analysis led to the estimation of the weights of the predictors. Figure 4b shows the weights of the coincident drought indices, while Fig. 4c shows the weights of the antecedent variables. The coefficients indicate how many standard deviations of BA anomalies change for every standard deviation unit change of the predictors. The average value of the coefficients for the coincident SPEI (i.e. the coefficients a(i) of Eq. 1) is −0.62 (and −0.66, considering only the 3 eco-regions where also antecedent conditions play a role), while the average for antecedent conditions (i.e. the coefficients b(i) of Eq. 1) is 0.46. That is, over most of Mediterranean Europe, the importance of the coincident drought is much larger than that of antecedent climate. Figure 5 shows the relationship between the CD coefficients and the latitude of the centroids of the eco-regions. There is a significant (*p* < 0.05) negative correlation between drought effect and latitude (*ρ* = −0.33), i.e., in northern regions drought seems to play a more prominent role than in southern areas.

## Discussion

In this work, we developed an ensemble of Multiple Linear Regression (MLR) models linking the summer (JJAS) Burned Area to the key drought drivers (estimated using the Standardized Precipitation Evapotranspiration Index, SPEI) in the Mediterranean region of Europe (Portugal, Spain, southern France, Italy and Greece). Specifically, we statistically investigated the dependence of summer fires on current-year drought conditions (proxies for the climatic factors that affect fuel dryness) and antecedent wet conditions (proxies for the climatic factors influencing total fuel and fine fuel structure, i.e., availability and connectivity).

This study represents a necessary step to address the question of whether climate change will increase fire activity in Mediterranean Europe. Climate change projections point to an increase in fire risk (see, e.g. refs 30, 46) but the effects of climate change on BA are not always obvious^42, 47^. For instance, increasing dry conditions could reduce BA by limiting vegetation growth and biomass (fuel availability).

The statistical analysis reported here shows that same-summer-drought conditions play a dominant role for fires in Mediterranean Europe. The link between Burned Area and coincident SPEI_3_ for July and August (when temperatures are usually highest) is strong in all Mediterranean Europe. Mediterranean ecosystems are characterized by a relatively high abundance of fine fuels that can dry out quickly during a single summer. A few areas in the southern Iberian Peninsula, France and Greece reveal higher correlations over longer accumulation periods, possibly related to the typical unimodal seasonal rainfall distribution for these regions, with a dry period extending from April to September.

The results of our statistical analysis are in agreement with other studies for the Mediterranean region, indicating that droughts promote larger fire activity (see, e.g. refs 8, 48 and references therein). The results also support, at least in some instances and with moderate statistical significance, the role played by antecedent climate which emerges in our study as well as in other regional analyses (see e.g. refs 16, 27, 28, 33, 49–52). Specifically, our analysis shows that in a few regions (in northern and eastern Spain and southern Italy), also antecedent wet conditions have an influence on fires, presumably because fuel productivity is controlled by previous climate conditions. For instance, antecedent wet conditions may allow for the fine-fuel to grow and ensure fuel continuity. Prior wet periods could thus favour the growth of specific fine fuels like the so called “Mediterranean scrub”. Antecedent climate may also promote fuel gaps to be filled within the landscape, resulting in an increased abundance and continuity of fuel load. Further work is required on this issue, possibly blending data analysis with process-based models.

The relatively strong link of BA with summer drought suggests that, overall, the mechanism by which drought affects BA is straightforward: warmer and drier summers lead to larger fires. However, the drought-fire relationship is more complex. Although we generally found a strong link between droughts and fires, droughts alone are not sufficient to predict BA across all regions. Interestingly, only a few zones (mainly in the southern part of the study domain) do not exhibit a statistically significant link between fire and drought, and a similar result has been obtained for Israel^42^. These results could provide an example of possible future evolution in transition areas on the border between Mediterranean and arid climates. On the other hand, the drought-BA relationship is stronger in northern regions. In other words, drought plays a more prominent role in northern (generally wetter and more productive) regions than in southern (drier) regions, possibly because in southern areas the vegetation is better adapted to water scarcity. This conclusion is consistent with the results obtained for vegetation-fire-climate relationships in Spain^20^.

The linear regression models developed here for each eco-region in Mediterranean Europe show high skill both in hindcast mode and in the out-of-sample leave-one-out test. These relatively simple regression models, linking drought indices with fire activity, can be used to estimate Mediterranean fire response to different climate change scenarios, assuming that climate-vegetation-human-fire interactions will not change significantly. The complex relationships between fires, climate variability, human activities and vegetation distribution may, however, limit the applicability of these findings to conditions which are very different from the current ones. Also, more complex analyses including other factors, such as fire suppression and fire ignition representations and wind condition, can provide additional understanding of the temporal evolution of the fire drivers.

Even with the limitations mentioned above, the results reported here indicate that summer fire risk may increase^29^ in Mediterranean Europe. In forthcoming decades, and especially for the northern Mediterranean regions, the direct effect of climate change in regulating fuel moisture (i.e., drought leading to larger fires) is expected to be dominant, relative to the indirect effect of antecedent climate on fuel load and structure (i.e., warmer/drier conditions limit fuel availability). Climate effects could become even stronger and overcome fire prevention efforts, implying that more fire management effort has to be planned in the near future. In the past few decades, the measured trend of BA in Mediterranean Europe has generally been negative^53, 54^, while drought conditions have generally been increasing^55^. These opposite trends suggest that management actions have so far counterbalanced the climatic trend. However, keeping fire management actions at the current level might not be sufficient to balance a future increase in droughts, thus calling for a rethinking of current management strategies^56^.

The results reported here were obtained by following a solid, simple and transparent statistical methodology that can also be applied to other areas. On the basis of these results, we developed an ensemble of parsimonious empirical models linking the summer Burned Area to the key climatic drivers. These simple models produce reliable out-of-sample predictions of the impact of climate variability on BA, and represent a necessary step to providing a substantial contribution to the development of a seasonal forecast system supporting fire management strategies. The ability to model the link between drought and forest fires is crucial to identifying key actions in adaptation strategies. Seasonal climate forecasts enable a more effective and dynamic adaptation to climate variability and change, offering an under-exploited opportunity to reduce the fire impact of adverse climate conditions. Seasonal drought prediction systems (see, e.g. ref. 57) could be integrated with the empirical models developed here for probabilistic drought-fire risk assessment.

## Data and Methods

### Fire and drought data

We obtained monthly BA (larger > 1 ha) data from the EFFIS^44^ dataset at NUTS3 level (2006 version; see http://ec.europa.eu/eurostat/web/nuts/ for more details) for Portugal, Spain, southern France, Italy and Greece, for the period 1985–2011 (see ref. 54 for more details).

We use the Standard Precipitation and Evaporation index (SPEI^39^) to estimate drought intensity. The SPEI transforms the climatic balance between precipitation and potential evapotranspiration over a specific period (usually from 1 to 12 months) into a Gaussian distribution with zero mean and unit standard deviation. Positive values indicate conditions of above-normal wet conditions, while negative values identify dry situations. The SPEI is based on monthly precipitation and potential evapotranspiration data (based on the FAO-56 Penman-Monteith estimation of potential evapotranspiration) from the Climatic Research Unit of the University of East Anglia (CRU version 3.22). The SPEI (v2.3) was obtained from http://sac.csic.es/spei/database.html.

The data are aggregated considering 43 eco-regions that we defined by combining the available fire information with the environmental zones defined by^58^ (see Supplementary Fig. S1). Fire data are available at NUTS3 level, and some of these divisions fell into more than one environmental zone. In these cases, NUTS3 were assigned to the zone that covers most of the NUTS3. We then aggregated the NUTS3 areas in eco-regions, retaining the division by countries in order to make it possible to transfer the results of this study to national policy makers and forest fire managers.

### Drought-fire model development

The MLR models link year-to-year changes in summer fires with current and antecedent drought indices:

1 $$\mathrm{log}\,[BA(i,t)]=a(i)\cdot CD(i,t)+b(i)\cdot AD(i,t)+\varepsilon (i,t)$$

where *BA*(*i*, *t*) is the predicted Burned Area in the *i* ^*th*^ eco-region and summer *t*; *a* and *b* are coefficients that vary spatially and represent the sensitivities of *BA* in each region to the Coincident Drought (*CD*) and the Antecedent Drought (*AD*) conditions (measured by the SPEI indicator), respectively; *ε* is a stochastic noise term that captures all other (neglected) factors that influence *BA* other than *CD* and *AD*. Drought conditions are measured by the SPEI indices aggregated in multi-month values, *SPEI*_*c*_(*τ*, *m*), where *c* is the accumulation time scale of 3, 6 and 12 months, *τ* is the time lag (in years, e.g. equal to 0 in case of coincident drought) and *m* is the month to which the index refers.

The procedure to develop this MLR model includes the following steps:

- We normalize the positively skewed BA variables by applying a log transformation (i.e. *Y* = log(*BA*));
- The time series of log(*BA*) and drought indices are linearly detrended to minimise the influence of slowly changing factors. Drought and log(*BA*) anomalies are then normalised by subtracting the time-series mean and dividing by the standard deviation. This standardization makes the MLR results for the different eco-regions comparable with each other.
- To identify the CD indicators, we (i) compute the correlation between log(*BA*) and *SPEI*_*c*_(*τ*, *m*), with c = (3, 6, 12), m = (0, 7), i.e., summer and previous spring months; (ii) calculate the significance of the individual correlations with coincident drought as the percentage of random coefficients that are lower than that obtained from the original (unshuffled) time series (i.e. one tailed hypothesis test); (iii) we test the p-values of previous step for multiple testing with a False Discovery Rate (FDR) test^45^; (iv) we seek the minimum correlation values among all the significant correlations calculated in the previous steps.
- To identify the AD indicators, we (i) calculate the partial correlation between log(*BA*) and antecedent drought variables referring to different months, ranging from 45 to 0 months before the end of the fire season, with the CD variable acting as control variable. For those regions where no CD is found, the standard correlation is tested instead of the partial correlation; (ii) calculate the significance of the individual partial correlations with antecedent drought variables as the percentage of random coefficients that are higher than that obtained from the original (unshuffled) time series (i.e. one tailed hypothesis test); (iii) we tested the p-values of previous step for multiple testing with an FDR test^45^; (iv) we seek the maximum correlation values among all the significant correlations calculated in the previous steps.
- Finally, for each eco-region we fit all the possible models with the selected predictors and retain only those models whose residuals satisfy the hypothesis of normality, zero autocorrelation and no trend. In regions where we need to deal with more than one variable, the models showing the lowest AIC (corrected for finite sample size^59^) are selected.

## Electronic supplementary material

Supplementary information
