# Supplementary material for: On the key role of droughts in the dynamics of summer fires in Mediterranean Europe
Source: Sci Rep. 2017 Mar 6;7:81. doi: 10.1038/s41598-017-00116-9 (PMC5427854; doi:10.1038/s41598-017-00116-9)
Supplement: Supplementary file 1 — Supplementary information [file 41598_2017_116_MOESM1_ESM.pdf]

On the key role of droughts in the dynamics of summer fires in Mediterranean Europe

Marco Turco[1,4,*], Jost von Hardenberg[2], Amir AghaKouchak[3], Maria Carmen Llasat[4], Antonello Provenzale[5], and Ricardo M. Trigo[6]

[1]Barcelona Supercomputing Center (BSC), Earth Sciences Department, Barcelona, 08028, Spain
[2]Institute of Atmospheric Sciences and Climate (ISAC), National Research Council (CNR), Torino, 10133, Italy
[3]Center for Hydrometeorology and Remote Sensing, Department of Civil and Environmental Engineering, University of California, Irvine, CA 92697, USA
[4]University of Barcelona, Department of Applied Physics, Barcelona, 08028, Spain
[5]Institute of Geosciences and Earth Resources (IGG), National Research Council (CNR), Pisa, 56124, Italy
[6]Instituto Dom Luiz (IDL), Faculdade de Ciências, Universidade de Lisboa, Lisboa 1749-016, Portugal
[*]Corresponding Author turco.mrc@gmail.com

Supplementary Figure 1. a) Eco-regions and relative codes used in this study and b) environmental zones defined by [1].

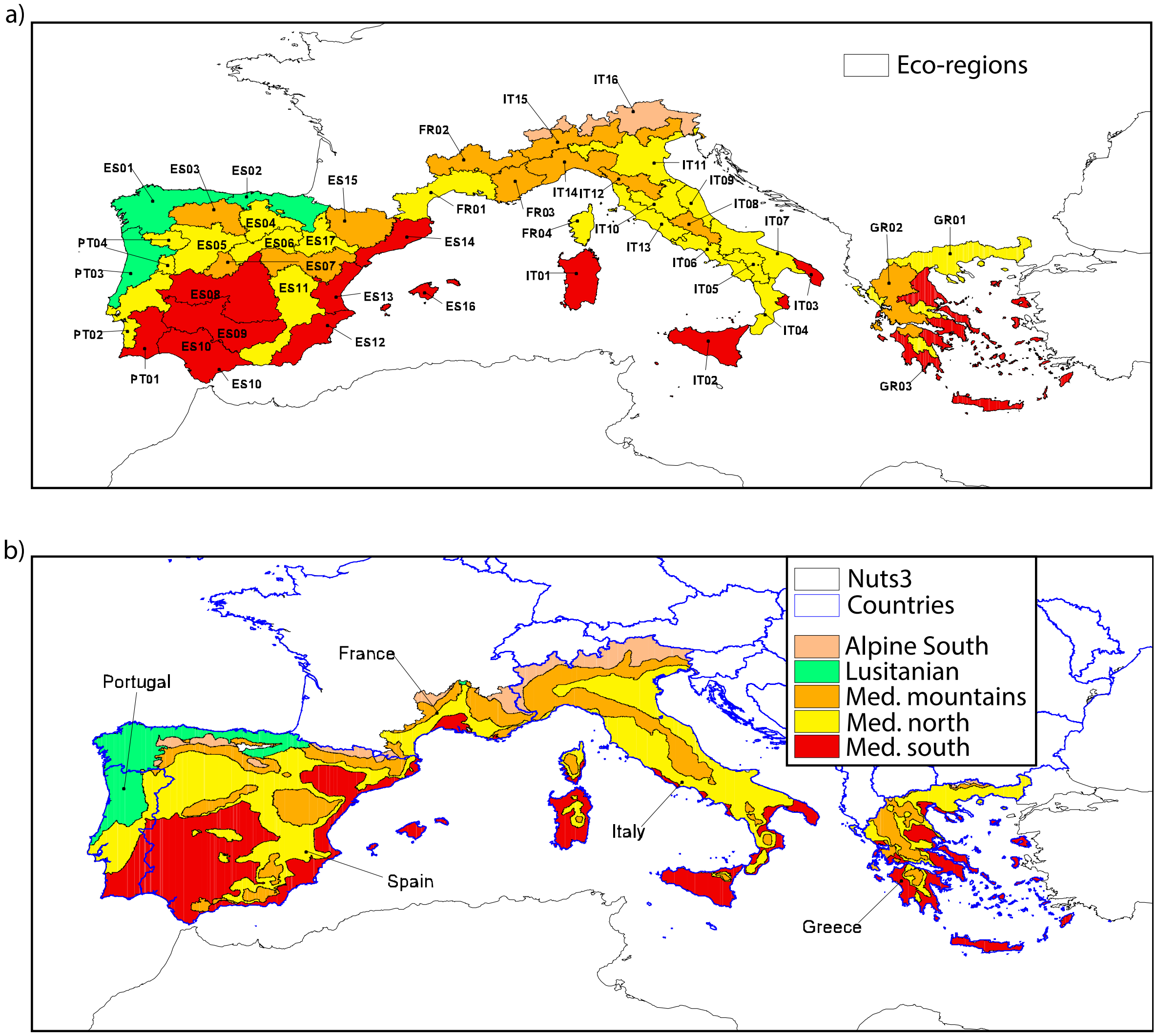

This figure is created with ArcView GIS Version 3.1 (http://www.esri.com/). The eco-regions used in this study are freely available for research purposes by applying to the corresponding author.

Supplementary Figure 2. Cross-correlations between detrended fire and drought variables.

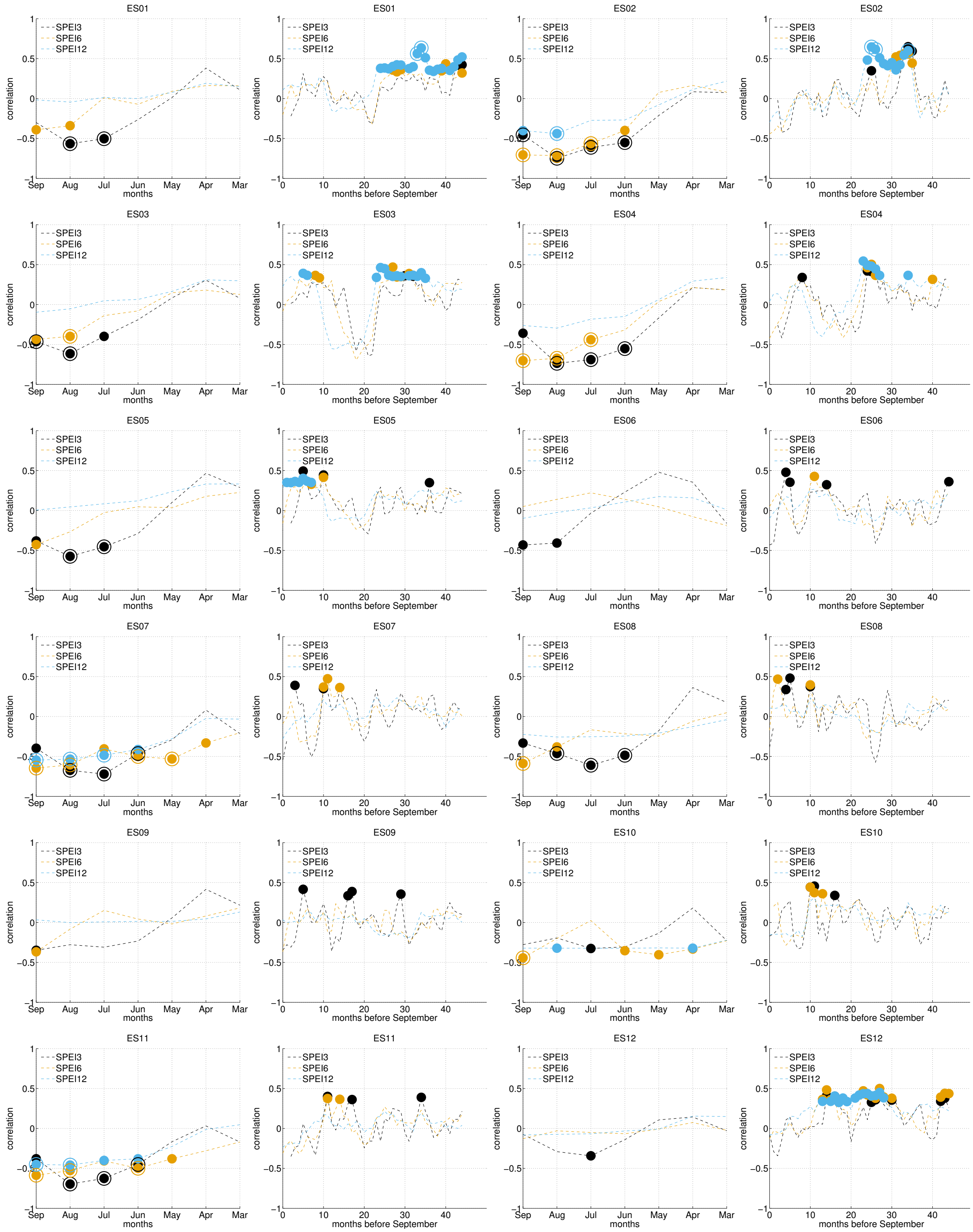

For each eco-regions, correlations (odd columns) among log(BA) and drought variables and partial correlations (even columns) among log(BA) and antecedent drought variables. For those regions where no coincident drought has been found, the standard correlation has been tested instead of the partial correlation. Filled circles indicate individually significant correlations (p-values < 0.05); open circles indicate correlations that are collectively significant with a FDR test [2].

Supplementary Figure 2. (Continuation)

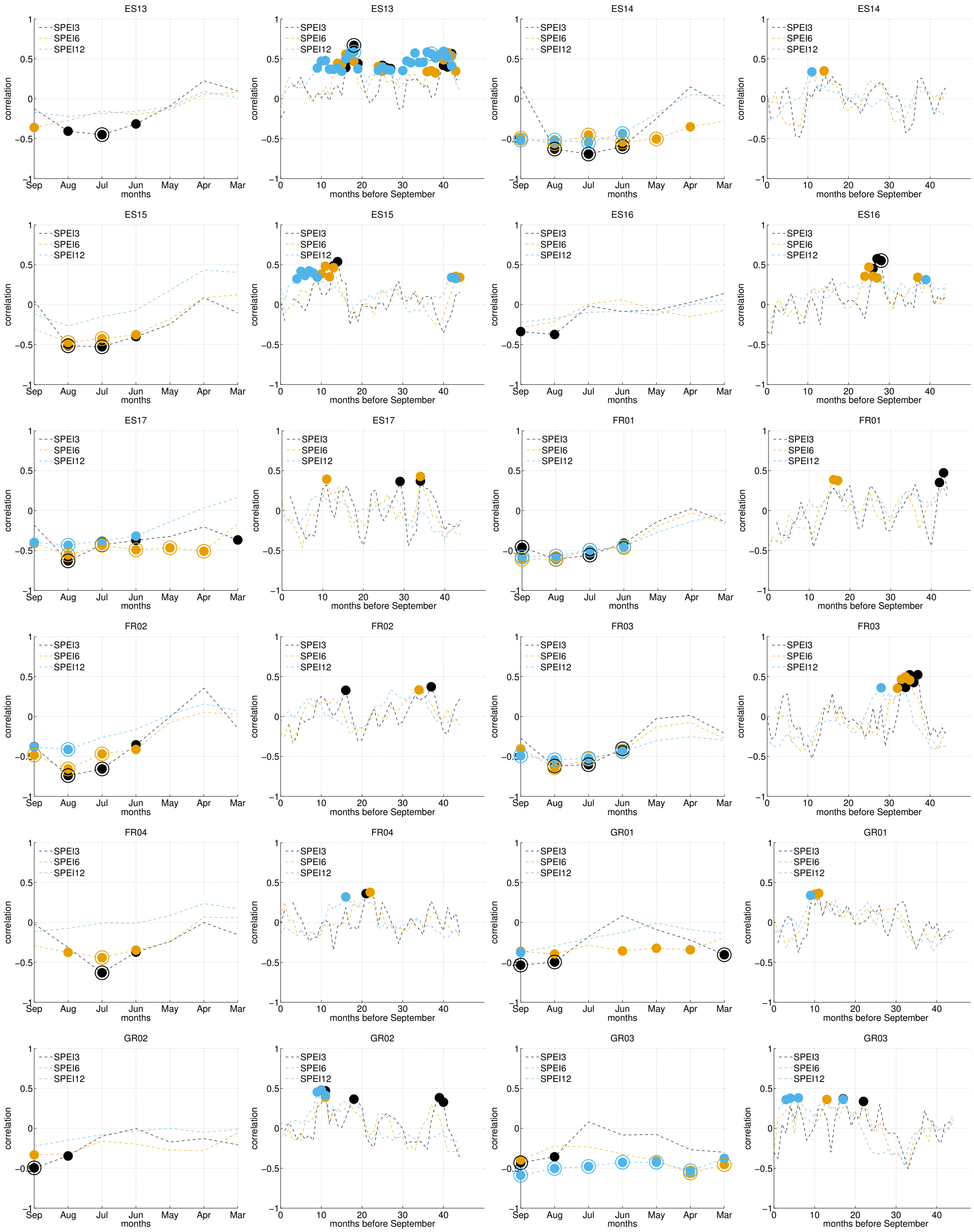

Supplementary Figure 2. (Continuation)

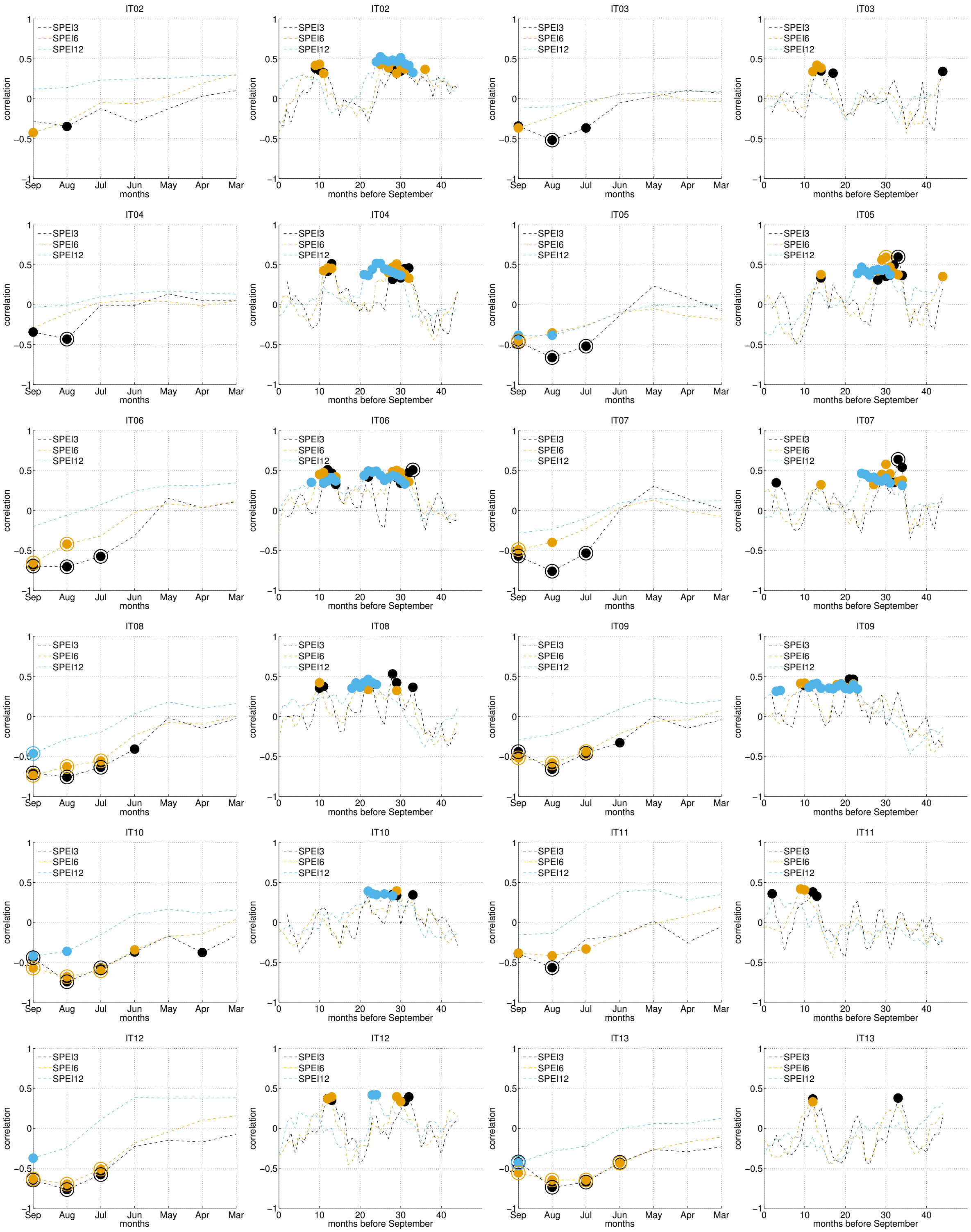

Supplementary Figure 2. (Continuation)

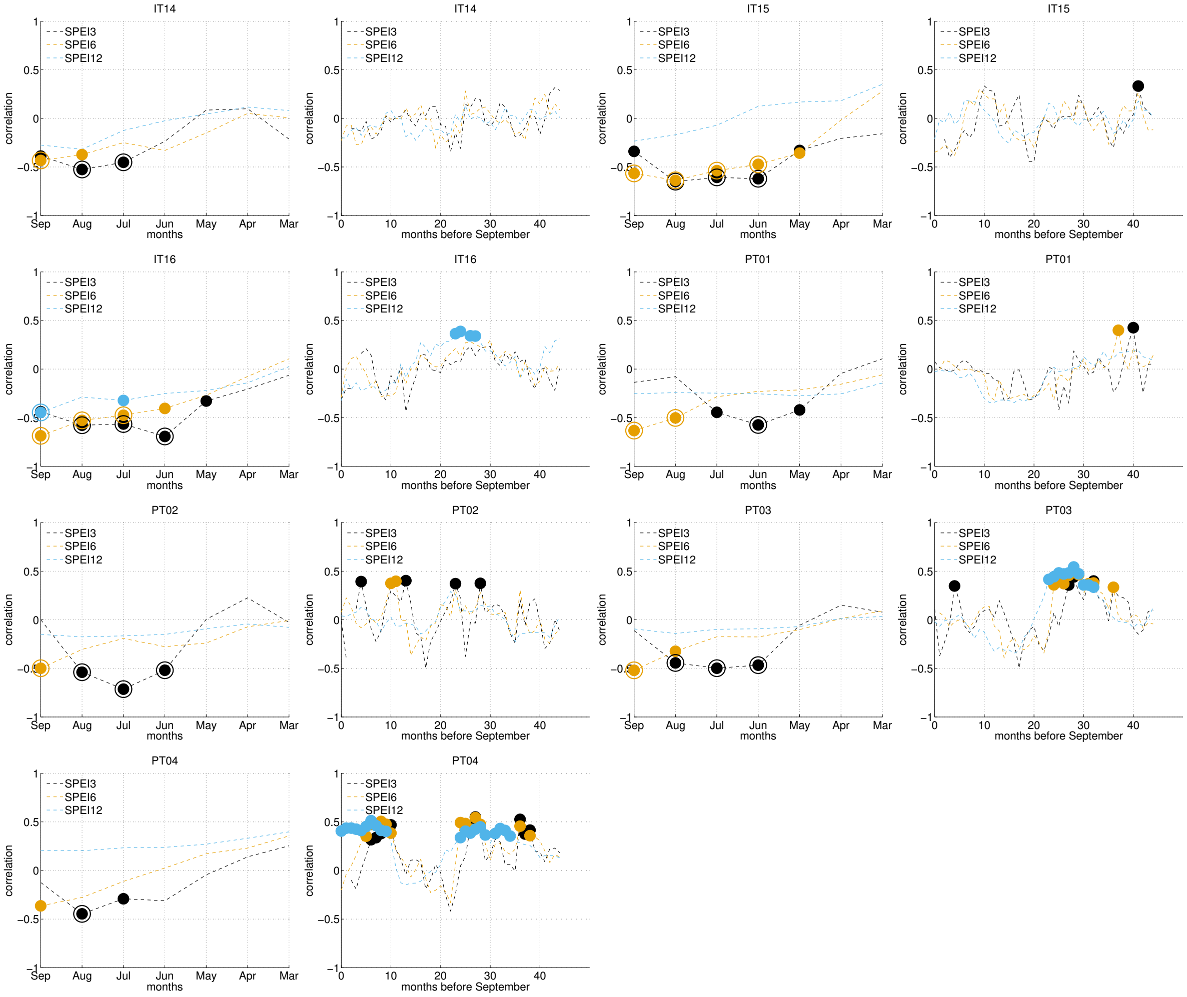

Supplementary References

[1] Metzger, M., Bunce, R., Jongman, R., Mücher, C. & Watkins, J. A climatic stratification of the environment of Europe. *Global ecology and biogeography* 14, 549–563 (2005).

[2] Ventura, V., Paciorek, C. J. & Risbey, J. S. Controlling the proportion of falsely rejected hypotheses when conducting multiple tests with climatological data. *Journal of Climate* 17, 4343–4356 (2004).
